# Out of Pocket Payment and Affordability of Medication for Geriatric Patients in Tehran, Iran

**Published:** 2019-06

**Authors:** Morvarid ZARIF-YEGANEH, Mona KARGAR, Arash RASHIDIAN, Aarefeh JAFARZADEH KOHNELOO, Kheirollah GHOLAMI

**Affiliations:** 1. Department of Clinical Pharmacy, School of Pharmacy, Tehran University of Medical Sciences, Tehran, Iran; 2. Research Center for Rational Use of Drugs, Tehran University of Medical Sciences, Tehran, Iran; 3. Department of Health Management and Economics, School of Public Health, Tehran University of Medical Sciences, Tehran, Iran; 4. Department of Epidemiology and Biostatistics, School of Public Health, Tehran University of Medical Sciences, Tehran, Iran

**Keywords:** Out-of-pocket, Affordability, Geriatric patients, Elderly, Medication cost

## Abstract

**Background:**

Considering the importance of high out-of-pocket (OOP) payment (OOPP), as a marker of health system performance, and affordability of medications in the elderly, this study was conducted to determine these issues.

**Methods:**

In this cross-sectional study, prescriptions of patients aged 65 yr or older from 5 university-affiliated pharmacies in Tehran, Iran were evaluated from Jan to Mar 2014. Prescriptions were selected from four insurance organizations. We used the prescriptions data regarding patients’ demographics and the prescribers as well as the sales data for OOP. Affordability was calculated by considering the daily salary of an unskilled worker.

**Results:**

Totally, 1467 prescriptions were analyzed. Mean age of patients was 73.89(6.66) yr. Mean (SE) of reimbursable and OOPP of the prescriptions were 203820 (10831) and 230252 (10634) IRR (Iranian Rials) respectively (equivalent to 81.6 (4.33) and 92.17 (4.33) US$ respectively). Subspecialists imposed higher expenditures for patients and insurance organizations. Patients referred to the ophthalmologists paid less OOP. Nearly 50% of the total prescription costs was paid as OOP. The mean OOPP was averagely equal to 1.41(0.065) daily salary. These prescriptions were unaffordable for 36.2% of patients.

**Conclusion:**

The OOPP was higher than the insurance goal of 30% for outpatients in Iran. More than one-third of elderly patients could not afford their single prescription. Due to the health consequences of the unaffordability of medications, corrective actions are needed by the insurance organizations and the health system.

## Introduction

Based on the 2015 published data, geriatrics constitute 8.5% of the worldwide population (1). The high increase in the geriatric population in Asia and the Pacific lead to one over 60 yr old in every four people by the year 2050 (2). In 2012, 8.26% of the Iran’s population were elderly and it is predicted that by 2022, Iran will have about 12 million elderly population (3).

By increasing the elderly population, the number of patients with polymorbidity increased reflectively. About 39% of the elderly patients experience 3 or diseases that are more chronic simultaneously compared to 15% of the non-elderly population (4). The importance of this high prevalence is the association between polymorbidity with polypharmacy (5). Because of increasing the number of medications, the medication costs increase. Unfortunately, in most Asian and developing countries patients’ out-of-pocket (OOP) comprise the main method in health care financing (6). OOP payment (OOPP) is the direct payment of patients for the costs of the healthcare services. This payment can be a part of the services that are partially covered by the insurance or might be uncovered by other payers’ (6). Medical OOPP was also defined as the payments including “coinsurance, copayments, deductibles, and medically related items and services not covered by insurance” (7). The OOP health care costs are considerably important since they lead to the poverty of 100 million patients annually (6).

The OOPP of patients with polymorbidity reaches more than 5% of their total income (8). This needs considerable attention since due to the lower income of the elderly (4) as a consequence of retirement or unemployment, the situation may result in poor adherence (8). The cost of the medications has been shown to affect the adherence in these patients (9–11).

A major concern for the governments is the affordability of the healthcare systems (12). The high OOPP can affect the affordability of treatment. In this subject, the role of insurance companies is also considerable.

Currently, there are several insurance organizations in Iran: Social Security Insurance Organization (SSIO), the Armed Forces Medical Services Insurance Organization (AFMSIO), Medical Services Insurance Organization (MSIO) (currently known as Health Care Insurance), Imam Khomeini Emdad Committee, and micro insurance funds (13). Despite various types of insurance policy, ordinarily, outpatients and inpatients payment proportion is expected to be 30% and 10% of the medicines expenditures respectively (14, 15). However, in recent years OOPP constituted over 45% of the annual medication cost (16). OOPP in several countries is approximately lower than Iran. For instance, in Australia (28%), South Korea (27%), Slovak Republic (26%), Sweden (22%), France, Luxembourg, Japan and Switzerland (17%), Germany (15%), Czech Republic (11%), Spain (6%), and USA (>30%)can be pointed (17, 18).

Due to the importance of the affordability of the medications, high OOPP and lack of adequate data regarding medication costs for elderly in Iran, we aimed to determine the prescription cost, OOPP and affordability of the prescription medications for the elderly patients.

## Materials and Methods

In this cross-sectional study, we included a sample of prescriptions from 5 public pharmacies affiliated with the faculty of pharmacy of Tehran University of Medical Sciences, Tehran, Iran. The study is part of a research project in which different aspects of pharmacotherapy for geriatrics were evaluated.

### Sampling and data collection

We aimed to collect 300 insured prescriptions of patients aged 65 yr or older from each pharmacy. To select the intended prescriptions based on the patients’ age, at the end of the month before the pharmacy sent the paper prescriptions to the insurance organizations, all of the prescriptions were screened consecutively and the included prescriptions were photographed. We included the prescriptions from the insurance organizations including the SSIO, AFMSIO, MSIO and rural insurance. The rural insurance fund is one of the funds of the MSIO. However, due to the differences, we evaluated these two separately. The proportion of the prescriptions from each insurance was aimed to be close to the population covered by these organizations at the study time. Based on the frequency of the geriatric prescriptions in each pharmacy, the duration of sampling lasted from Jan to Mar 2014.

On each dispensed prescription in the pharmacies routinely a unique code is printed. This made the linkage between the data of the prescription photographs with the sales data of the pharmacies possible. We used the sales data of pharmacies’ belonging to the collected prescriptions to record the items in the prescriptions, number of each item, the cost per each item, the reimbursements, the costs of the uncovered items, and the OOPP per prescription. Other data including patients’ demographics (age and sex) and the physicians’ specialties were added using the prescriptions photographs. Data were analyzed using SPSS 24 software (Chicago, IL, USA) in order to calculate the OOPP and determine the associated factors.

### OOP calculation

The OOP consisted of the summation of the costs of the uncovered items, the coinsurance, and the dispensing fee. Additionally, the remainder of the difference between the costs of each medication (brands or generics) with the lowest-price generic medicine covered by the insurance organization was also added.

### Evaluation of affordability

We applied a previously used definition based on the minimum daily salary of an unskilled worker (15). This value is determined annually by the Social Security Organization of Iran. At the time of our study, the minimum daily salary was 162,375 IRR (equals to 6.5 US$). We divided the OOPP of patients for each prescription by the mentioned value to find the number of daily salaries that each patient needed to spend on his medications. If the prescriptions’ expense needed the patient to spend more than one day of the salary it was defined as unaffordable.

### Statistical Methods

Descriptive statistics were applied to explore the data. Nonparametric Kruskal Wallis test was used to compare the mean of quantitative variables. Post-hoc tests were performed by controlling the type-1 error. To report a correlation between variables Spearman correlation coefficient was also calculated.

### Ethics approval

The study was approved by the Ethics Committee of the Tehran University of Medical Sciences (TUMS). Consent form was not applicable for this study.

## Results

We collected 1512 prescriptions of patients older than 65 yr from five pharmacies. However, 45 prescriptions were excluded due to the unavailability of the details of the prescription costs. Therefore, we evaluated 1467 prescriptions belonged to the elderly. Mean (SD) age of patients was 73.89 (6.66) yr and mean number of items per prescription was 3.4 (Table 1).

**Table 1:** Characteristics of the patients and prescribers

***Characteristic***	***Prescriptions N (%)***
Sex	
Female	693 (47.4)
Insurance	
SSIO	868(59.2)
MSIO	418(28.5)
AFMSIO	113 (7.7)
Rural	67 (4.6)
Prescribers	
GP	461 (31.4)
Medical Resident	108 (7.4)
Specialist	609 (41.5)
Subspecialist	283 (19.3)
Dentist	6 (0.4)
Prescribers specialty	
Internal medicine	343 (23.5)
Cardiology	206 (14.1)
Ophthalmology	78 (5.3)
Neurology	64 (4.4)
Others	309 (21.1)

AFMSIO: Armed Forces Medical Services Insurance Organization, MSIO: Medical Services Insurance Organization, SSIO: Social Security Insurance Organization, GP: general practitioner

### Total and reimbursable prescription costs

**Fig. 1:**
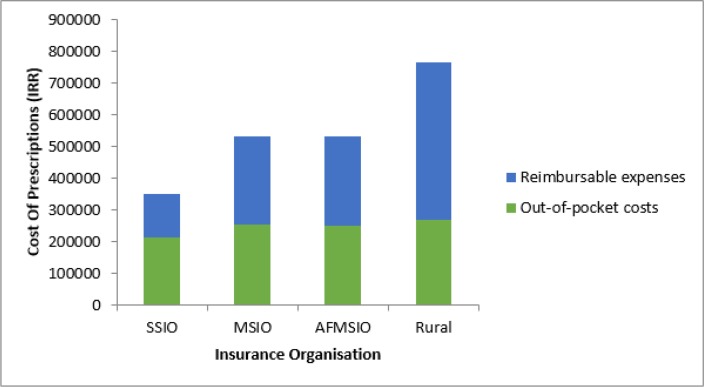
Total costs, reimbursable expenses and out-of- pocket payments of patients for prescriptions in different insurance organizations

The mean (SE) of total costs and the reimbursable of the prescriptions were 434072 (16792) and 203820 (10831) IRR respectively (equivalent to 173.7 (6.72) and 81.59 (4.33) US$ respectively). Figure 1 shows the total cost of the prescriptions in terms of OOPP and reimbursable expenses. The mean reimbursable prescription expenses were significantly different among insurance companies (*P*-value<0.001). Based on the Post-hoc tests it was significantly lower in the prescriptions covered by SSIO (all *P*-value <0.001).

There was not a significant difference between the mean reimbursable expenses and the mean total costs of the prescriptions between men and women (*P*-value=0.731) and (*P*-value=0.429) respectively. Moreover, no significant correlation was found between the total costs and age of the patients (r=0.052, *P*-value=0.053).

### OOPP

The mean (SE) OOPP of patients was 230252 (10634) (range 0 to 5154400) IRR (equivalent to 92.17 (4.25) (range 0 to 2063.41) US$). As shown in Table 2, the OOPP of patients was significantly different among prescriptions covered by different insurance companies (*P*-value<0.001).

**Table 2:** Costs of the prescriptions covered by different insurance organizations

***Cost (IRR)***	***Insurance Organizations***				***P-value***
	***SSIO***	***MSIO***	***AFMSIO***	***Rural***	
OOP Mean (SE)	213067 (13888)	253570 (18509)	252357 (47272)	271088 (45929)	≤0.001
Reimbursable expenses, Mean (SE)	136380 (5530)	276740 (23344)	278128 (38509)	493615 (152007)	≤0.001
Mean (SE) number of daily salary to pay for the prescriptions	1.31 (0.08)	1.56 (0.11)	1.55 (0.29)	1.66 (0.28)	0.001
OOP/mean number of items in prescription, Mean (SE)	73819 (5648)	85140 (8436)	89258 (16335)	122647 (23859)	0.001
OOP/total cost, Mean (SE)	0.52 (0.007)	0.48 (0.01)	0.43 (0.02)	0.43 (0.03)	≤0.001

AFMSIO: Armed Forces Medical Services Insurance Organization, MSIO: Medical Services Insurance Organization, SSIO: Social Security Insurance Organization, OOP: out-of-pocket

At the time of the study 1US$ was equal to almost 24980 IRR

Post-hoc tests showed that prescriptions covered by MSIO had significantly higher average OOPP compared with those covered by SSIO (*P-*value<0.001). The mean (SE) of OOPP per each item was 80475 (4445) (range 0 to 2570200) (equivalent to 32.21 (1.78) (range 0 to 1028.90) US$). Comparing this values among different insurance companies showed a significant difference among them (*P*-value=0.001). In fact, patients insured by rural insurance and MSIO paid significantly higher OOP per item compared to those with SSIO coverage (*P*-value=0.013, *P-*value=0.022).

The mean (SE) of proportion of OOPP to the total cost was 0.497 (0.005). This proportion was also significantly different among insurance companies (*P*-value<0.001), and based on the Post-hoc tests, it was significantly higher in SSIO (all *P*-values <0.001).

There was not a significant difference between the mean OOPP between the prescriptions of men and women (*P*-value=0.238). Additionally, no significant correlation was found between the OOPP and age of the patients (r=0.047, *P-*value=0.076).

### Calculation of affordability

Mean OOPP was averagely equal to 1.41(0.065) daily salary of the patients. Additionally, in paired comparisons, only SSIO and MSIO insurance were significantly different in terms of affordability (*P*-value=0.001) and significantly less mean number of daily salary was needed to be paid by patients covered with the SSIO compared with MSIO to obtain their medications. We found a positive and significant correlation between the number of items in the prescriptions with the mean number of daily salary needed to be paid for the medications (r=0.402, *P*-value <0.001).

Additionally, among our patients 529 (36.2%) patients had to pay more than one daily salary to purchase their prescription medications, which means that the medications were unaffordable for them. The results of the chi-square test showed that in SSIO, the frequency of these cases was lower (Pearson Chi-Square= 9.61 and *P-*value=0.022).

### The role of the prescribers in the prescription costs

A chi-square test of independence was performed to examine the relationship between insurance organizations and the academic degree of prescribers. The relation between these variables was significant (Pearson Chi-Square= 55.29 and *P-*value<0.001). Patients covered with SSIO insurance were less likely visited by the subspecialists (Table 3). Additionally, comparisons of costs were conducted for the prescribers with various academic degrees (Table 4). The OOPP and the reimbursable expenses were significantly different among prescribers with different academic degrees (*P*-value <0.001). For both costs, Post-hoc tests showed that except for the comparison between general practitioners (GPs) and medical residents (*P*-value=0.351, *P*-value=0.648), all the other pairwise comparisons were significantly different (all *P*-value <0.04). In fact, the subspecialists imposed more expenditures to patients as well as the insurance organizations compared to the other prescribers. Average OOP per each prescription item was also significantly different among prescribers with various academic degrees (*P*-value<0.001). GPs had the lowest and the subspecialists had the highest average (all *P-*values<0.005). However, the differences in the proportion of OOPP to the total cost was not significant (*P*-value =0.074). When the affordability of the prescriptions was analyzed, we found that in paired comparisons, the mean number of daily salary needed to be paid for the prescriptions was significantly different between the prescribers and was higher for the prescriptions written by subspecialists compared to all the others (all *P*-values<0.002). The results for the comparison of prescriptions’ cost among various medical specialties are summarized in Table 5. The OOPP and reimbursable expenses were significantly different among different specialties (*P-*value<0.001).

**Table 3:** Number and percentages of prescription by prescribers with different academic degree in different insurance organizations

		***Academic degree of prescribers N(%)***				***Total***
		***GP***	***Medical Resident***	***Specialist***	***Subspecialist ***	
Insurances Organizations	SSIO	309 (35.8)	70 (8.1)	340 (39.4)	145 (16.8)	864 (100)
	MSIO	124 (29.8)	23 (5.5)	173 (41.6)	96 (23.1)	416 (100)
	AFMSIO	28 (24.8)	4 (3.5)	55 (48.7)	26 (23.0)	113 (100)
	Rural	0 (0.0)	11 (16.4)	40 (59.7)	16 (23.9)	67 (100)

AFMSIO: Armed Forces Medical Services Insurance Organization, MSIO: Medical Services Insurance Organization, SSIO: Social Security Insurance Organization, GP: general practitioner

**Table 4:** Costs of the prescriptions prescribed by prescribers with different academic degree

***Cost (IRR)***	***Academic Degree of Prescribers***				***P-value***
	***GP***	***Medical Resident***	***Specialist***	***Subspecialist***	
OOP Mean (SE)	132601 (14589)	238186 (50703)	231386 (13308)	383856 (33530)	≤0.001
Reimbursable expenses, Mean (SE)	95951 (3836)	153283 (16330)	232030 (21501)	338116 (27789)	≤0.001
Mean (SE) number of daily salary to pay for the prescriptions	0.82 (0.09)	1.46 (0.31)	1.42 (0.08)	2.36 (0.21)	≤0.001
OOP/number of items, Mean (SE)	40693 (4244)	109463 (29776)	84404 (5556)	125761 (13818)	≤0.001
OOP/Total cost, Mean (SE)	0.502 (0.008)	0.504 (0.022)	0.497 (0.008)	0.483 (0.013)	0.074

GP: general practitioner, OOP: out-of-pocket

At the time of the study 1US$ was equal to almost 24980 IRR

**Table 5:** Costs of the prescriptions by different prescribers

***Cost (IRR)***	***Prescribers***						***P-value***
	***GP***	***Ophthalmology***	***Internal Medicine***	***Cardiology***	***Neurology***	***Others***	
OOP, Mean (SE)	132601 (14589)	68542 (8966)	316905 (24117)	325483 (35727)	471052 (84312)	207210 (17772)	≤0.001
Reimbursable expenses, Mean (SE)	95951 (3836)	65767 (12057)	316801 (21057)	228693 (16857)	214609 (23786)	255371 (41985)	≤0.001
Mean (SD) number of daily salary to pay for the prescriptions	0.816 (0.089)	0.422 (0.055)	1.951 (0.148)	2.004 (0.220)	2.901 (0.519)	1.276 (0.109)	≤0.001
OOP/number of drug, Mean (SD)	40693 (4244)	29012 (3642)	114844 (11867)	88584 (11215)	142385 (26007)	96437 (11232)	≤0.001

GP: general practitioner, OOP: out-of-pocket

At the time of the study 1US$ was equal to almost 24980 IRR

Post-hoc tests showed that patients referring to the ophthalmologists paid less OOP (all *P-*values<0.007) while visiting by internists, cardiologists and neurologists imposed more costs to the patients (all *P*-values<0.006). The same results were noted for reimbursable prescription expenses (all *P*-values<0.001). Average OOPP per each item, was significantly different between prescriptions by various prescribers (*P-*value<0.001), GPs and ophthalmologists had the lowest meanwhile internists, cardiologists and neurologists had the highest mean (all *P-*values<0.004).

In paired affordability comparisons, the mean number of daily salary needed to be paid for the prescriptions written by internists, cardiologists and neurologists were significantly higher than other specialists. In contrast, the mean number of daily salary needed for the prescription payment was the lowest for the prescriptions by ophthalmologists (all *P*-values <0.007).

### The most expensive medicines

The most expensive drugs in the prescriptions were Dipherelin^®^ prefilled syringe, oxaliplatin 100 mg vial, oxaliplatine 50 mg vial, albumin 20% vial and Spiriva^®^ Handihaler respectively.

## Discussion

OOP health care expenses have been used as a marker of health system performance (19). In this study nearly 50% of the total costs of the prescriptions were paid by patients as OOP. Although the government planned to reduce the OOPP to 30% by 2008, it was reported that the health OOP was as high as 55% in 2009 (20). This high share of OOP in the Iranian health care system was also reported previously (21). The importance of the current study is partially due to the fact that the cost of treatment has a role in receiving adequate care or precluding treatment. This is not only the case in the developing countries, but also affect patients in countries such as the US (12) and Australia (22). Additionally, the elderly pay higher OOP for health (23) even after adjustments for sex, marital status and insurance (4). Moreover, in these patients, the OOP on medications accounts for a considerable “financial burden”(24). Since nearly one-third of the total medical OOP costs in the elderly belongs to the medications (24, 25).

We did not find any association between patients’ age with OOPP or total costs of the prescriptions. In contrast, it was reported that in patients older than 90 yr the medication burden decreased considerably (26). However, this was attributed to the alteration of the “risk benefit profile” of some medications for this age group (26). Moreover, we did not find a significant association between sex and OOPP. While a study on data from 1992 to 2000 showed that the elderly women in the US paid higher OOP for the prescription medication compared to men (24).

In this study, the specialists constituted the major group of prescribes and among them internists were the main group. None of the patients with rural insurance were visited by GPs. This was because the patients in the rural area have to be visited by primary care physicians in their area and then if a visit by a specialist is needed they will be referred. We found that subspecialists imposed higher expenditures to patients and insurance organizations compared to the other prescribers. This led to the significantly higher mean days of salary needed to be paid as OOP for their prescriptions. Additionally, average OOP per each item in prescriptions was the highest for the subspecialists. This can be due to the higher expenses of the specialized medicines since the most expensive medicines in this study were specialized medicines.

When different insurance organizations were compared, in the prescriptions covered by SSIO, the OOPP (compared with MSIO), mean OOPP per each item (compared with rural and MSIO) and mean reimbursable expenses were significantly lower. This finding can be to some extent due to the lower percentage of subspecialists among the prescribers of these prescriptions. Although the mean proportion of OOPP to the total cost was significantly higher in SSIO, patients with this insurance had a significantly better affordability compared with MSIO. This contrast can be explained by considering the lower total cost of prescriptions covered by SSIO as shown in Table 2 and Fig. 1.

One of the important aspects of health policy is the affordability of health care for geriatrics (19). However, we found that patients had to pay averagely 1.41 d of salary for obtaining their prescription. In fact, prescriptions were unaffordable for 36.2% of the study population. This is also the case in several other countries. For example, in Malaysia with a similar methodology for the assessment of affordability, many patients could not afford their medication since one-month treatment with fluoxetine needed 26 d wages and for amlodipine and simvastatin needed between 5–7 d wages (27).

### Limitations

We used OOPP for the calculation of the affordability. However, due to the unavailability of the written orders for daily doses of all of the medications/prescriptions, it was not clear that the prescriptions contained one-month medicines or more. All of the pharmacies were located in Tehran. Therefore, the number of patients referred to these pharmacies with rural insurance was not adequate for the study sampling. Therefore, we included more SSIO prescriptions instead of the rural prescriptions in the pharmacies with a very limited number of the latter prescriptions. Additionally, there might be differences in the affordability of treatment of different diseases such as a large variability found in the affordability of treatment for pneumonia and malaria in Sudan (28). However, we could not separate different diseases and their treatment costs and affordability in this study due to the unavailability of patients’ diagnosis in the pharmacies. Another limitation of the present study was that our judgment regarding the OOPP was based on a single prescription of patients. However, for elderly patients with several chronic conditions and prescriptions written by different prescribers, this calculation may underestimate the current status of affordability and OOPP.

## Conclusion

The OOPP was higher than the insurance goal of 30% for outpatients in Iran. Additionally, more than one-third of elderly patients in this study could not afford their single prescription. Due to the health consequences of the unaffordability of medications, corrective actions are needed by the insurance organizations and the health system.

## Ethical considerations

Ethical issues (Including plagiarism, informed consent, misconduct, data fabrication and/or falsification, double publication and/or submission, redundancy, etc.) have been completely observed by the authors.
